# Diabetic Retinopathy Is a Predictor of Progression of Diabetic Kidney Disease: A Systematic Review and Meta-Analysis

**DOI:** 10.1155/2022/3922398

**Published:** 2022-04-29

**Authors:** Mansi Gupta, Indu Ramachandra Rao, Shankar Prasad Nagaraju, Sulatha V. Bhandary, Jayanti Gupta, Ganesh T. C. Babu

**Affiliations:** ^1^Center of Application and Research in India, Carl Zeiss India (Bangalore) Pvt. Ltd, Bangalore, Karnataka, India; ^2^Department of Nephrology, Kasturba Medical College, Manipal, Manipal Academy of Higher Education, Manipal, Karnataka, India; ^3^Department of Ophthalmology, Kasturba Medical College, Manipal, Manipal Academy of Higher Education, Manipal, Karnataka, India; ^4^Nishkash Consulting, New Delhi, India

## Abstract

**Methods:**

A systematic search was conducted on PubMed, Embase, and the Google scholar for eligible studies through September 2021. The quality of selected articles was assessed using JBI checklist. Higgins and Thompson's *I*^2^ statistic was used to see the degree of heterogeneity. Based on degree of heterogeneity, fixed or random effects model was used to estimate pooled effect using inverse variance method. Results were expressed as hazard ratios and odds ratios with 95% CIs.

**Results:**

After scrutinizing 18017 articles, data from ten relevant studies (seven prospective and three retrospective) was extracted. DR was significantly associated with DKD progression with a pooled HR of 2.42 (95% CI: 1.70–3.45) and a pooled OR of 2.62 (95% CI: 1.76–3.89). There was also a significant association between the severity of DR and risk of progression of DKD with a pooled OR of 2.13 (95% CI: 1.82–2.50) for nonproliferative DR and 2.56 (95% CI: 2.93–.33) for proliferative DR.

**Conclusion:**

Our study suggests that presence of DR is a strong predictor of risk of kidney disease progression in DKD patients. Furthermore, the risk of DKD progression increases with DR severity. Screening for retinal vascular changes could potentially help in prognostication and risk-stratification of patients with DKD.

## 1. Introduction

The global burden of diabetes mellitus (DM) has been steadily rising over the past few decades. The International Diabetes Federation (IDF) predicts an increase in the number of patients with diabetes to 643 million by 2030 and to 784 million by 2045 [1]. An estimated 30–50% of patients with diabetes will develop DKD during their lifetime [2]. DKD is, at present, the leading cause of end-stage kidney disease (ESKD) worldwide and causes significant morbidity, mortality, and healthcare burden [3, 4]. Indeed, much of the cardiovascular death in diabetes appears to be closely related to the development of kidney disease [5, 6]. The prediction of DKD progression in patients with type 1 and type 2 DM represents an important clinical and public policy challenge.

DKD is often not recognized in the initial stages due to lack of routine screening for microalbuminuria, especially in low-resource settings. Clinicians are heavily reliant on serum creatinine and urine dipstick, both of which are flawed screening tools, and there is a paucity of validated biomarkers that can help in early detection of chronic kidney disease (CKD) [7]. There is also considerable heterogeneity in the natural history of DKD which makes staging and prognostication challenging. While some patients progress through the classically described five stages of diabetic nephropathy (DN), some with microalbuminuria regress to normoalbuminuria and do not progress, whereas some normoalbuminuric patients develop progressive renal function decline, without a preceding microalbuminuria stage [8–12]. Thus, early recognition of those at risk of progression to ESKD is the need of the hour to enable timely initiation of nephroprotective therapies to prevent or slow worsening of kidney disease and thereby attenuate DKD-associated mortality and healthcare burden.

DR is one of the microvascular complications in diabetes and approximately affects about 30% of patients with diabetes [13]. There is remarkable homology between the eye and the kidney in terms of developmental, structural, and pathophysiological pathways and it is, therefore, rather unsurprising that DKD is often closely associated with DR [14]. In patients with diabetes, glycemic and blood pressure control reduces the incidence and progression of both DR and DKD, suggesting a common pathogenesis of these two complications [15–21].

Clinically, DR is screened with fundoscopy, which is a relatively noninvasive, inexpensive method [22, 23]. It can be routinely performed during outpatient screening for chronic complications of diabetes. The presence of established DR strongly suggests DKD as the cause of kidney disease in a diabetes patient with renal dysfunction and epidemiological studies have found that DR may be a good screening tool for recognition of DN [24–30]. However, the role of DR as a predictor of progression to ESKD has not been clearly elucidated. Thus, in this systematic review and meta-analysis, we sought to pool data from available studies and obtain a more reliable estimate of the effect of DR on the risk of progression of DKD in patients with DM.

## 2. Methods

### 2.1. Search Strategy

A systematic search of PubMed, Embase, and Google Scholar was performed by two independent reviewers (M.G. and T.C.B.) for articles studying the association between DR and progression of DKD in September 2021. Using MeSH terms as well as the free words, the search strategy included the following: [((diabetic retinopathy stage OR retinopathy stage) AND (risk factor OR prognostic factor OR predict OR etiology) AND (nephropathy stage OR diabetic nephropathy stage OR DKD stage) AND (diabetic nephropathy progression OR nephropathy progression)]. The last search date was September 30, 2021.

### 2.2. Selection Criteria

The inclusion criteria for articles were as follows: (1) longitudinal studies (prospective and retrospective); (2) studies including patients with DM (irrespective of type of DM) and presumed or biopsy-proven DKD; (3) presence of DR defined according to well-validated scales, such as the Early Treatment Diabetic Retinopathy Study severity scale; and (4) studies reporting progression of DKD. Studies with no full text, cross-sectional design, and insufficient data to compute hazards ratio (HR) (or odds ratio) for DKD progression were excluded. The rigorous eligibility criteria were strictly followed by two independent reviewers (M.G. and T.C.B.) to control for publication bias. Any disagreements in the inclusion of a paper were resolved by a third researcher (I.R.R.).

### 2.3. Data Extraction and Quality Assessment

Using standardized forms, data on year of publication, first author's name, country where study was undertaken, study setting, follow-up periods, sample size, definitions of the exposure (DR) and outcome (ESKD), baseline demographic data including average age of the participants, duration of diabetes, average blood pressure, HbA1c, average eGFR values, and the reported effect estimates (HR or OR with 95% CIs) were extracted from the eligible studies.

The Joanna Briggs Institute (JBI) critical appraisal checklist for cohort studies was used to assess the quality of the selected articles [31]. The tool contains eleven items. The evaluation scores ranged from 0 to 11, with <3 defined as “low quality,” 3–6 as “moderate quality,” and >6 as “high quality.” The JBI quality scores of included articles are provided in Table 1.

### 2.4. Data Synthesis and Analysis

Results were expressed as hazard ratios and odds ratios with 95% CIs. The degree of between-study heterogeneity was estimated using the Higgins and Thompson's *I*^2^ statistic, with *I*^2^ of >75% indicating considerable heterogeneity. Pooled effect estimates for DR and DKD progression, as well as for DR severity and risk of DKD progression, were computed using the inverse variance method. A fixed effects model was used when between-study heterogeneity was low, while a random effects model was used if heterogeneity was high. A sensitivity analysis was performed to ascertain robustness of the obtained results. To ascertain publication bias, a funnel plot was constructed by plotting the standard errors of the studies against the corresponding HR. All analyses were performed using R version 4.1.1.

## 3. Results

### 3.1. Literature Search Results

**Figure 1:**
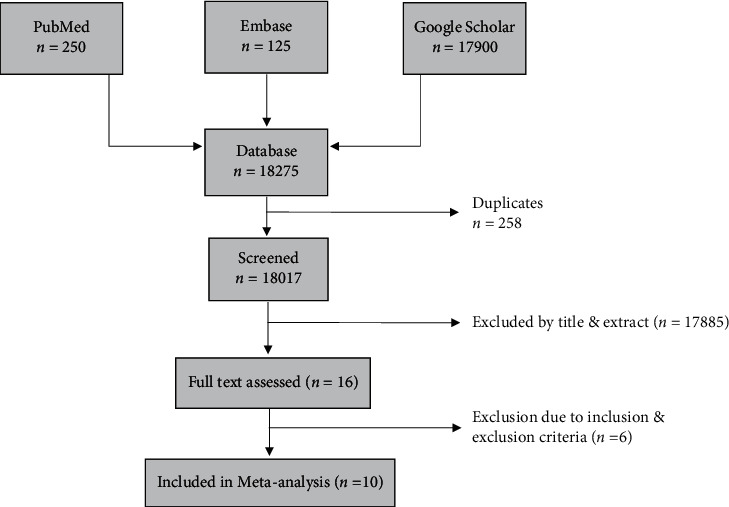
Systematic review and meta-analysis flow diagram.

A total of 18,275 articles were identified after a rigorous search on PubMed, Embase, and Google Scholar. Out of these 18,275 articles, 258 articles were identified as duplicates. The remaining 18017 articles were screened based on the abstracts and titles, separately, by two independent reviewers. Any disagreements on the inclusion of a paper were resolved by the third researcher. A full-text assessment was conducted and finally 10 articles were included in the meta-analysis. The article selection process has been summarized in Figure 1.

### 3.2. Study Characteristics

The relevant characteristics of the selected publications are summarized in Tables 1 and 2. A total of 14,355 patients across the 10 studies are included. All included studies were judged to be of high quality, with a score of seven or more as per the JBI checklist.

### 3.3. DR and DKD Progression

**Figure 2:**
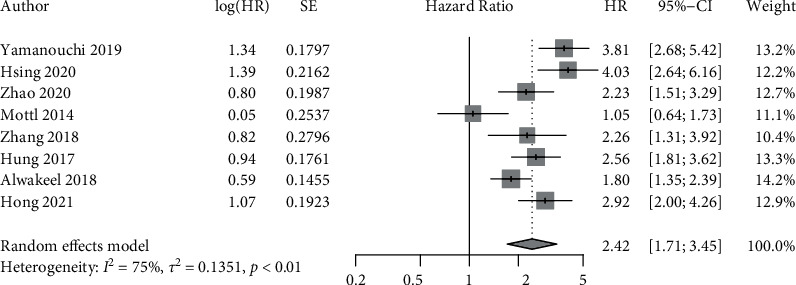
Forest plot depicting the pooled hazard ratio (HR) for DR as a predictor of DKD progression.

**Figure 3:**
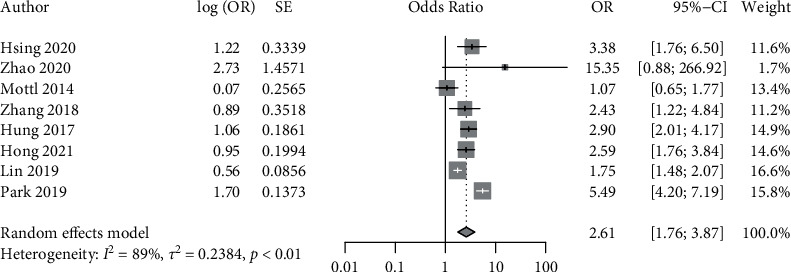
Forest plot depicting the pooled odds ratio (OR) for DR as a predictor of DKD progression.

Of the ten studies, six reported results using both HR and OR, two reported HR alone, and two reported OR alone. Therefore, results were analysed, and pooled estimates were computed as HR and OR both, separately. Since the *I*^2^ statistic indicated considerable heterogeneity for both HR (*I*^2^ = 75.2%, *p* < 0.01) and OR (*I*^2^ = 89%, *p* < 0.01), a random effects model was used. The obtained results are presented in the forest plots below (Figures 2 and 3). Both pooled estimates indicated a significant association between presence of DR and DKD progression, with pooled HR of 2.42 (95% CI: 1.70–3.45) and pooled OR of 2.62 (95% CI: 1.76–3.89).

### 3.4. Severity of DR and DKD Progression

Four studies reported the association of severity of DR and DKD progression. Data as OR was available in three studies (Hsing 2020, Lin 2019, and Park 2019), while Yamanouchi et al. reported results as HR and raw data to compute OR was not available [32, 33, 40, 41]. Thus, data from three studies was pooled (Table 3), while the results of the Yamanouchi 2019 study are described separately below. Our meta-analysis found a pooled OR of 2.13 (95% CI: 1.82–2.50) for NPDR and 3.56 (95% CI: 2.93–4.33) for PDR.

Yamanouchi et al. also reported a similar stepwise increase in DKD progression with increasing DR severity with an HR of 1.35 (0.49–3.76) for mild NPDR, 2.89 (1.42–5.86) for moderate NPDR, 5.00 (2.63–9.52) for severe NPDR, and 5.32 (2.89–9.78) [32].

### 3.5. Sensitivity Analysis and Publication Bias

Sensitivity analysis was performed after excluding (a) studies that did not have biopsy-proven DKD patients and (b) studies that were retrospective. There was no significant change in the obtained results with pooled HR of 2.76 (95% CI: 1.26–6.04) and OR of 1.79 (95% CI: 1.04–3.09), respectively.

**Figure 4:**
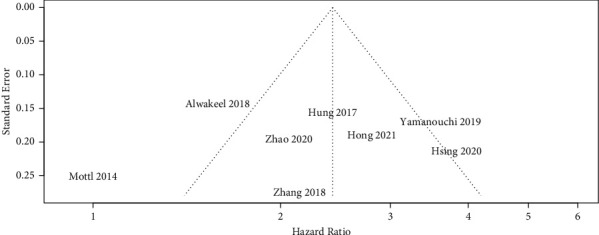
Funnel plot depicting possible publication bias.

The funnel plot (Figure 4) demonstrated an asymmetric distribution of studies indicating a possible publication bias.

## 4. Discussion

This systematic review included ten longitudinal studies with a pooled sample size of 14,355. Meta-analysis of data from the included studies found that the presence of DR was significantly associated with DKD progression, with a pooled hazard ratio of 2.42 (95% CI: 1.70–3.45). The obtained results were consistent even when pooled estimates were reported in terms of odds ratio (OR 2.62, 95% CI:1.76–3.89). Sensitivity analysis excluding studies with presumed DKD (rather than biopsy-proven cases) and that excluding retrospective studies also yielded similar results, indicating robustness of our findings. All the included studies in our review found a significant association between DR and ESKD, except the one by Mottl et al. [35]. There are two possible factors that may explain the lack of association in the Mottl et al. study. Firstly, patients with serum creatinine of >1.5 mg/dL were excluded from this study. Hence, very few patients (<2.5%) developed ESKD during the study period and this low event rate may have influenced the results. Secondly, patients were grouped as having no/mild DR versus moderate/severe DR, rather than as having no DR versus having DR. It is important to note that this study did find that DR was associated with a nearly twofold increase in risk of doubling of serum creatinine and an increase in incident macroalbuminuria.

Furthermore, we found that the severity of DR was also significantly associated with DKD progression. There was an increase in DKD progression risk as severity of DR increased, with a pooled OR of 2.13 (95% CI: 1.82–2.50) for NPDR and 3.56 (95% CI: 2.93–4.33) for PDR. Yamanouchi et al. reported a similar relationship with HR of 1.35 (0.49–3.76) for mild NPDR, 2.89 (1.42–5.86) for moderate NPDR, 5.00 (2.63–9.52) for severe NPDR, and 5.32 (2.89–9.78) [32].

Apart from DR, the association of other retinal vascular signs and kidney disease has also been studied. Studies have found that retinal vascular diameters, derived from fundus photographs, were significantly associated with CKD. Yau et al. reported that retinal arteriolar narrowing was associated with incident CKD, while Yip et al. found that retinal venular widening was a predictor of risk of CKD [23, 42]. Lee et al. evaluated retinal perfusion status in patients with diabetes using fluorescein angiography and found that nonperfusion of more than ten-disc areas was associated with a sixfold risk of CKD progression [43].

While the association of DR and presence of DKD is already clearly established, it is unclear whether presence of DR (and its severity) is also associated with DKD progression and our study specifically answers this question. Given the unpredictable natural course of DKD, the ability to identify patients at high risk of progression of DKD using DR findings would be an invaluable clinical tool and could potentially guide the nephrologist to individualize therapies tailored to an individual's future risk of ESKD. Tests to ascertain presence (and severity) of DR are noninvasive and economical, thereby making implementation of routine DR screening practicable.

A recent meta-analysis by Guo et al. found an association between DR and incidence of cardiovascular (CV) disease and CV mortality in patients with diabetes [44]. Therefore, a fundus examination in a patient with diabetes is necessary to not just detect DR early and prevent consequent vision loss but may also have a role in risk-stratification of patients in terms of renal and CV outcomes. Such an approach could not only improve patient outcomes but could also improve the efficiency and cost-effectiveness of healthcare.

This evidence provides strong support to build prediction models that can ascertain an individual's risk of ESKD, based on fundus examination. Over the last few years, there have been major breakthroughs in automated and semiautomated retinal analysis systems using artificial intelligence (AI) [45]. Although their large-scale use is yet to become a reality, AI prediction models for DKD progression could soon become an integral part of a digital health management platform; some institutions are working on such AI prediction models which could ease the burden of healthcare workers and revolutionize the field of medicine [45, 46].

To the best of our knowledge, this is the first meta-analysis to formally evaluate this association of DR and DKD progression. Although the consistency of the association across different measures of effect and sensitivity analyses indicates credibility of our findings, our meta-analysis is limited by the small number of relevant studies that were obtained in the systematic review and the considerable heterogeneity between the studies. Most of the included studies were retrospective and thus carry a risk of confounding. Likewise, in most of the studies, diagnosis of DKD was presumptive and not biopsy-proven. A majority of the included studies reported association of DR and DKD progression risk in type 2 DM and so it is unclear whether the findings of this meta-analysis can be extrapolated to patients with type 1 DM.

## 5. Conclusion

Our meta-analysis found that DR is a strong predictor of determining progression of CKD in patients with DKD. This suggests that screening for retinal vascular changes could potentially help in prognostication and risk-stratification of patients with DKD. The severity of DR also can predict progression of DKD. Well-conducted large prospective studies are needed to confirm the findings of this study.

## Figures and Tables

**Table 1:** Characteristics of selected studies.

Study	Type of study	Follow-up period	Type of diabetes	Aim of study	Basis for DR diagnosis/classification	Basis for DKD diagnosis	Study definition of DKD progression	Results	JBI score
Yamanouchi et al. [32] 2019, Japan (*N* = 232)	Retrospective	5.7 years (median)	Type 2	(1) To evaluate the association between clinical findings in the retina and pathological lesions in kidney biopsy specimens and (2) to quantify the risk for ESKD, according to the severity of diabetic retinopathy, in patients with type 2 diabetes and biopsy-proven DKD	Review of medical records on retinal/fundus Examination DR classification: ICDR scale	Biopsy-proven DKD	Progression to ESKD (defined as initiation of any hemodialysis, peritoneal dialysis, or renal transplantation, or death from uremia)	**HR (95% CI) of ESKD risk for each DR stage, relative to no DR** Mild NPDR: 1.35 (0.49–3.76) Moderate NPDR: 2.89 (1.42–5.86) Severe NPDR: 5.00 (2.63–9.52) PDR: 5.32 (2.89–9.78)	10
Hsing et al. [33] 2020, Taiwan (*N* = 1329)	Retrospective	1.97 years (mean)	Type 2	To evaluate the renal disease progression (ESKD and CKD) in patients with type 2 diabetes and with/without DKD according to DR severity	Fundus photographs analysed by deep learning models and confirmed by ophthalmologist DR classification: ICDR scale	Presumed DKD	Progression to ESKD (defined as initiation of any hemodialysis, peritoneal dialysis, renal transplantation or death from uremia)	**HR (95% CI) of ESKD risk for each DR stage, relative to no DR** Mild NPDR: 2.35 (1.01–5.43) Moderate NPDR: 5.96 (2.99–11.89) Severe NPDR: 2.79 (1.16–6.75) PDR: 6.98 (2.22–21.94)	9
								**HR (95% CI) of CKD for each DR stage, relative to no DR for subjects without CKD initially (n** **=** **841):** Mild NPDR: 3.46 (0.92–12.98) Moderate NPDR: 8.75 (2.74–27.92) Severe NPDR: 5.73 (1.64–20.04) PDR: 14.21 (1.55–130.67)	
Zhao et al. [34] 2020, China (*N* = 91)	Retrospective	15 months (median)	Type 2	(1) To classify DR as mild, moderate, or severe nonproliferative, or proliferative by artificial intelligence and an ophthalmologist, in Chinese patients with biopsy-confirmed DKD (2) To determine whether the severity of DR at the time of biopsy can predict progression to ESKD and (3) To characterize the relationship between DR and DKD in patients with DKD	Digital fundus photographs analysed by the lesion-aware deep learning system (RetinalNET) DR classification: early treatment diabetic retinopathy study severity scale (ETDRS)	Biopsy-proven DKD	Progression to ESKD (defined as eGFR<15 ml/min/1.73 m^2^, or the use of renal replacement therapy)	**HR (95% CI) of ESKD risk for DR, relative to no DR:** 2.23 (1.51–3.29)	10
								**ESRD (n,%) for each stage of baseline DR:** no DR: (0,0%), mild: (1, 9.1%), moderate: (13, 30.2%), severe: (6, 46.2%), PDR: (5, 55.6%)	
Mottl et al. [35] 2014, USA (*N* = 3210)	Prospective	4 years (mean)	Type 2	To evaluate specificity of DR for renal versus CV disease	Fundus photographs evaluated by trained graders DR classification: ETDRS final diabetic retinopathy severity scale	Presumed DKD	Progression to ESKD (defined as eGFR<15 ml/min/1.73m2 or if a participant was on dialysis or received renal transplantation)	**HR (95% CI) of ESKD risk for moderate/severe DR, relative to no/mild DR:** 1.05 (0.64, 1.73)	10
Zhang et al. [36] 2018, China (*N* = 141)	Retrospective	19 months (median)	Type 2	To identify whether DR was associated with the progression of DKD in patients with T2DM and biopsy-proven DKD	Ophthalmoscopy, equivocal diagnosis was validated with optical coherence tomography and fundus colour photography DR classification: NR	Biopsy-proven DKD	Progression to ESKD (defined as eGFR<15 mL/min/1.73 m^2^ or the initiation of renal replacement therapy)	**HR (95% CI) of ESKD risk for DR, relative to no DR:** 2.264(1.309–3.917)	10
Hung et al. [37] 2017, Taiwan (*N* = 1330)	Prospective	2.9 years (median)	Types 1 and 2	To study if longer diabetes duration, DR, and a diagnostic model were associated with less favourable renal outcomes, cardiovascular events and all-cause mortality in nonbiopsied patients with DKD	Fundoscopy/digital fundus photography examination DR classification: background, preproliferative, and proliferative changes	Presumed DKD	Progression to ESKD (defined as initiation of hemodialysis, peritoneal dialysis, or renal transplantation)	**HR (95% CI) of ESKD risk for DR, relative to no DR**: 2.56 (1.81–3.62)	9
Alwakeel et al. [38] 2011, Saudi Arabia (*N* = 621)	Retrospective	9.9 years (mean)	Type 2	To evaluate the pattern and changes in GFR over time and investigate the potential risk factors associated with enhanced loss of renal function and all-cause mortality among Saudis with type 2 diabetes and nephropathy	NR	Presumed DKD	Drop in KDIGO GFR category	**HR (95% CI) of CKD progression for DR, relative to no DR:** 1.8 (1.3–2.3)	10
Hong et al. [39] 2021, USA (*N* = 1759)	Retrospective	14.2 years (median)	Types 1 and 2	To examine the association between retinopathy and kidney disease in persons with diabetes in the community-based atherosclerosis risk in communities (ARIC) study	Fundus photographs assessed by masked graders. DR classification: early treatment diabetic retinopathy study severity scale	Presumed DKD	Incident ESKD [defined by linkage to the US renal data system (USRDS)]	**HR (95% CI) of ESKD risk for DR, relative to no DR**: 2.92 (2.00–4.26)	9
Lin et al. [40] 2019, Singapore (*N* = 4050)	Prospective	<1 year	Types 1 and 2	To evaluate the characteristics of CKD patients, with or without DR, and examine the relation between DR and its severity on the decline rate in the eGFR in stages 1–5 CKD patients	Dilated fundus examination and fluorescein angiography DR classification: no apparent signs of DR: normal; microaneurysms, hard exudates, intraretinal hemorrhages, venous beading, or prominent intraretinal microvascular abnormality: early stage, nonproliferative DR (NPDR); and retinal or optic disk neovascularization, vitreous hemorrhage, or preretinal hemorrhage: late stage, proliferative DR (PDR)	Presumed DKD	eGFR decline by more than 5 mL/min/1.73 m^2^/year	**OR (95% CI) of DKD progression for DR, relative to no DR**: 1.75 (1.48–2.07)	10
Park et al. [41] 2019, Korea (*N* = 1592)	Retrospective	5.6 years (mean)	Type 2	To assess the value of DR severity to predict renal dysfunction and albuminuria progression in type 2 DM patients	Slit-lamp examination, indirect ophthalmoscopy and/or fluorescein angiography DR classification: no DR, NPDR and PDR	Presumed DKD	Decline in GFR category accompanied by ≥25% eGFR drop OR sustained decline in eGFR of more than 5 mL/min/1.73 m^2^/year	**OR (95% CI) of DKD progression for DR, relative to no DR** NPDR: 4.05 (2.993–5.488) PDR: 9.293 (6.569–13.146)	10

**Table 2:** Demographic and clinical characteristics of participants.

First author	Gender male (%)	Age, mean (yrs)	Duration of diabetes, median (years)	BMI, mean (kg/m^2^)	History of smoking (%)	Hypertension (%)	History of CVD (%)	HbA1C, median (%)	Baseline mean eGFR (mL/min/1.73 m^2^)	24 h Proteinuria/UPCR, median(g/g)	UACR, median(mg/g)
Yamanouchi [32]	78	59	14	24	61	NA	24	7.3	39	NA	1400
Hsing [33]	52.9	63.28 ± 12.75	NA	25.5	NA	44.12	16.3	7.9	NA	NA	NA
Zhao [34]	75	51	10	25.19	48.5	79.1	NA	7.7	58.02	3.6	NA
Mottl [35]	61.2	61	10.75	32.4	NA	NA	36	8.3	89.3	NA	19.6
Zhang [36]	68.8	52.6	6.2	25.3	45.9	84.6	NA	7.36	65.9	4.55	NA
Hung [37]	62.3	64.2	>8 years	25.5	13.8	67.1	27.4	7.6	33.7	0.92	NA
Alwakeel [38]	47.2	66.9	15.4	28.6	NA	84.4	NA	NA	50.35	NA	NA
Hong [39]	48.8	63.4	8.9	31.7	59.9	65	38.8	NA	85.9	NA	5.8
Lin [40]	56.3	65.76	NA	25.96	26.7	91.3	NA	7.57	46.7	NA	NA
Park [41]	47.2	57.9	9.6	24.8	NA	48.5	NA	8.1	76.75	NA	24.2

**Table 3:** Pooled HR across DR stages.

Study	HR for NPDR (95% CI)	HR for PDR (95% CI)
Hsing 2020	3.28 (1.70–6.33)	5.77 (1.74–19.17)
Lin 2019	1.57 (1.29–1.91)	2.18 (1.71–2.78)
Park 2019	4.05 (2.99–5.49)	9.29 (6.57–13.15)
**Pooled estimate** 95.6% = *I*^2^	2.13 (1.82–2.50)	3.56 (2.93–4.33)
